# Oxidative stress mediated arterial dysfunction in patients with obstructive sleep apnoea and the effect of continuous positive airway pressure treatment

**DOI:** 10.1186/1471-2466-12-36

**Published:** 2012-07-23

**Authors:** Maria Del Ben, Mario Fabiani, Lorenzo Loffredo, Licia Polimeni, Roberto Carnevale, Francesco Baratta, Marco Brunori, Fabiana Albanese, Teresa Augelletti, Francesco Violi, Francesco Angelico

**Affiliations:** 1 Department of Internal Medicine and Medical Specialities, Sapienza University, Rome, Italy; 2 Department of Public Health and Infectious Diseases, Division of Internal Medicine C, Policlinico Umberto 1, Viale del Policlinico 155, 00161, Rome, Italy

## Abstract

**Background:**

Several studies suggest an increase of oxidative stress and a reduction of endothelial function in obstructive sleep apnoea syndrome (OSAS). We assessed the association between OSAS, endothelial dysfunction and oxidative stress. Further aim was to evaluate the effect of nasal continuous positive airway pressure (nCPAP) on oxidative stress and arterial dysfunction.

**Methods:**

We studied 138 consecutive patients with heavy snoring and possible OSAS. Patients underwent unattended overnight home polysomnography. Ten patients with severe OSAS were revaluated after 6 months of nCPAP therapy. To assess oxidative stress in vivo, we measured urinary 8-iso-PGF2α and serum levels of soluble NOX2-derived peptide (sNOX2-dp). Serum levels of nitrite/nitrate (NOx) were also determined. Flow-mediated brachial artery dilation (FMD) was measured to asses endothelial function.

**Results:**

Patients with severe OSAS had higher urinary 8-iso-PGF2α (p<0.001) and serum NOX2 and lower NOx. A negative association was observed between FMD and OSA severity. Apnea/hypopnea index was significantly correlated with the indices of central obesity and with urinary 8-isoprostanes (r=0.298, p<0.001). The metabolic syndrome (t=-4.63, p<0.001) and urinary 8-isoprostanes (t=-2.02, p<0.05) were the only independent predictors of FMD. After 6-months nCPAP treatment, a significant decrease of serum NOX2, (p<0.005) and urinary 8-iso-PGF2α (p<0.01) was observed, while serum NOx showed only a minor increase. A statistically significant increase of FMD was observed (from 3.6% to 7.0%).

**Conclusions:**

The results of our study indicate that patients with OSAS and cardiometabolic comorbidities have increased oxidative stress and arterial dysfunction that are partially reversed by nCPAP treatment.

## Background

Obstructive sleep apnoea syndrome (OSAS) is a common nocturnal disorder characterized by the presence of repetitive apnoea and hypopnoea during sleep, daytime sleepiness and cardiopulmonary dysfunction. Patients with OSAS experience recurrent episodes of cessation of breathing which expose the cardiovascular system to cycles of hypoxia, exaggerated negative intrathoracic pressure and arousals [1].

The majority of OSAS patients show the cluster of metabolic and non-metabolic cardiovascular risk factors of the metabolic syndrome and it has also suggested that OSA may be a manifestation of metabolic syndrome (MS) [2-4].

Several studies have provided evidence supporting an increase of oxidative stress in OSAS [5-10]. Oxidative stress is characterized by an imbalance between oxidant and antioxidant mechanisms, in which many different enzymatic and non-enzymatic antioxidants take place. Recently, it has been postulated that intermittent hypoxia can induce inflammation and that the development of inflammation in response to hypoxia may be clinically relevant. [11]. Previous studies have also demonstrated that total nitrate and nitrite (NOx) production is lower in OSAS patients than in controls [12,13]. Both oxidative stress and inflammation are major components in the initiation and development of endothelial dysfunction, which is widely accepted as an early marker of atherosclerosis. Flow-mediated, brachial artery vasodilation (FMD) is a well established marker of endothelial function, as the result of endothelial release of NO [14-16]. Endothelial function, which is the result of a reduction in NO bioavailability, is markedly reduced in patients with moderate/severe OSAS [17-23]. Chronic low-grade inflammation, oxidative stress, metabolic abnormalities and endothelial dysfunction in OSAS could accelerate atherogenesis. However, so far, the biological mechanisms which may explain the association of OSA with endothelial dysfunction are still under debate.

Nasal continuous positive airway pressure (nCPAP) is first-line therapy for OSAS to reduce daytime sleepiness and improve cardiovascular and metabolic outcomes [24]. Previous studies have demonstrated a beneficial effect of nCPAP on several markers of oxidative stress in patients with OSAS [25-29]. An improvement of arterial dysfunction after nCPAP therapy has been also demonstrated [12,30-33].

Aim of our study was to assess the association between OSAS, endothelial dysfunction and oxidative stress in a sample of patients with different severity of OSAS. Further aim was to evaluate the effect of nCPAP on FMD and the levels of markers of systemic oxidative stress in patients with severe OSAS after using nCPAP for six months.

## Methods

### Patients

The study group consisted of 138 consecutive patients who were referred to our metabolic outpatients clinic because of suspected metabolic disorders with heavy snoring and possible OSAS. All patients had a complete clinical and biochemical work up, including polysomnography (PSG), as part of routine clinical examination.

Written consent was obtained from all subjects before the study and the study conforms to the ethical guidelines of the 1975 Declaration of Helsinki. The research protocol was approved by the University Department of Experimental Medicine and Pathology scientific board in 2004. According to the Hospital Ethical Committee guidelines, ethical approval was not required since the study was observational and did not include the use of new drugs and/or new experimental treatments. To be eligible for the study, patients had to fulfil the following criteria: no history and clinical signs of heart failure, autoimmune disease, acute inflammatory disease, or any severe disease shortening life expectancy, such as diagnosed cancer, chronic liver disease, severe renal disease.

Arterial blood pressure was measured on the right arm with the subjects in a sitting position and after a 5-min rest, using a mercury sphygmomanometer: the average of two measurements, 1 min apart, was considered. Waist circumference, height and weight were recorded with subjects wearing light clothing, without shoes and body mass index (BMI) was calculated as weight (Kg) divided by height (m^2^). MS was diagnosed following the International Diabetes Federation criteria [34]. Diabetes was diagnosed according to the WHO criteria. Subjects taking insulin or oral antidiabetic drugs were considered to have diabetes.

### Flow-mediated vasodilatation

Ultrasound assessment of endothelial dependent and independent FMD of brachial artery was investigated according to the recently reported guideline [16]. Briefly, the study was performed in a temperature-controlled room (22°C) with the subjects in a resting, supine state between 8 a.m. and 10 a.m. after at least a 8-hour fasting; brachial artery diameter was imaged using a 7.5-Mhz linear array transducer ultrasound system (Siemens) equipped with electronic callipers, vascular software for two-dimensional imaging, colour and spectral Doppler, and internal electrocardiogram; the brachial artery was imaged at a location 3–7 cm above the antecubital crease; to create a flow stimulus in the brachial artery, a sphygmomanometric cuff was placed on the forearm; the cuff was inflated at least 50 mmHg above systolic pressure to occlude artery inflow for 5 min; all vasodilatation measurements were made at the end of diastole; FMD was expressed as a change in post-stimulus diameter evaluated as a percentage of the baseline diameter.

### Blood sampling protocol

Fasting venous blood samples were taken in the supine position on the morning after performing polysomnography and stored at −80°C until assay. A final morning fasting blood sample was obtained after using CPAP treatment for a period of six months.

Subjects underwent routine biochemical evaluation including fasting total and HDL-cholesterol, triglycerides, glucose and insulin. Serum total cholesterol, HDL-cholesterol and triglycerides were measured by an Olympus AN 560 apparatus using an enzymatic colorimetric method. LDL-cholesterol levels were calculated according to the Friedwald formula. Plasma insulin levels were assayed by commercially available radioimmunoassay. The homeostasis model assessment (HOMA-IR) was used to estimate insulin resistance using the formula: glucose (mmol/L) x [insulin (mU/L)/22.

A colorimetric assay kit (Tema Ricerca, Italy) was used to determine nitric oxide metabolites nitrite and nitrate (NOx) in the serum. Intra-assay and interassay coefficients of variation were 2.9% and 1.7% respectively.

Serum levels of soluble NOX2-derived peptide (sNOX2-dp) were detected by ELISA method as previously described [35]; intra-assay and inter-assay coefficients of variation were 5.2% and 6%, respectively. Values are expressed as pg/ml.

Urinary 8-iso-prostaglandin F2α (8-iso-PGF2α) was measured by a previously described and validated enzyme immunoassay method [36]. Intra-assay and inter-assay coefficients of variation were 2.1% and 4.5%, respectively.

### Polysomnography (nocturnal recording)

Patients underwent unattended overnight home PSG using a overnight home sleep recording (Embletta, PDS; Medcare, Reykjavik, Iceland). The device recorded nasal and oral airflow, chest and abdominal movements, and pulse oximetry. The sleep recordings were downloaded to a computer and scored by a principal investigator. A minimum of 5 hours of recording was accepted to be adequate for scoring. The presence and severity of apnea was assessed based on the number of apnoea/hypopnoea episodes per hour of sleep (apnoea/hypopnoea index AHI). Apnea was defined as continuous cessation of airflow for more than 10-s and hypopnoea was defined as reduction of airflow for more than-10 s with oxygen desaturation of ≥4% and arousal. OSAS was defined as an AHI of ≥5. Patients were categorized into four subgroups according to OSA severity, as follows; normal AHI <5 events/h; mild OSA, AHI <5 to <15 events/h; moderate OSA, AHI <15 to <30 events/h; severe OSA, AHI ≥30 events/h.

Patients had overnight home pulse oximetry monitoring with a transcutaneous fingertip sensor connected via cable to an OhmedaBiox 3700 pulse oximeter (Louisville, CO). The mean haemoglobin oxygen saturation level (SaO_2_) in total sleep time was also calculated.

Patients with severe OSAS underwent a full-night nCPAP titration study at home using an automated pressure setting device. The first consecutive 10 patients with severe OSAS who adhered to nCPAP treatment over a period of six months were revaluated.

Adherence to nCPAP was defined as nCPAP use for at least 4 hours per night and 5 days per week.

### Statistical analysis

Statistical analysis was performed by using the SPSS statistical software version 11.0 for Windows (SPSS,Inc.,Chicago.Illinois). All variables were tested for normal distribution prior to analyses. Data are expressed as the mean ± SD for continuous variables. The correlation between variables was analysed with the Pearson and the Spearman tests. Student’s t-test for unpaired data was used for the comparison of mean values. Group comparisons were performed by use of analysis of variance and test for linear trend in One-way ANOVA. Proportions and categorical variables were tested by the χ^2^–test and by the 2-tailed Fisher’s exact method when appropriate. Multiple linear regression analysis was performed to determine the independent predictors of FMD. All *P*-values are two-tailed; a *P*-value of less than 0.05 was considered statistically significant.

## Results

Out of the 138 patients who performed overnight polysomnography, 47 had a primary snoring and 91 had a positive polysomnography for OSA: 61 had mild/moderate and 30 severe OSA.

Clinical and metabolic characteristics of patients with OSA by severity and control snorers are reported in Table 1. A strong positive association was observed between OSA severity and the indices of central obesity, i.e. body mass index (p=<0.01) and waist and hip circumferences (p<0.001). In addition, a positive association was observed with serum insulin and HOMA-IR level and with urinary 8-iso-PGF2α concentration. As compared to non-OSAS, patients with severe OSAS had statistically significant higher urinary 8-iso-PGF2α and higher serum sNOX2-dp and lower NOx, although not at a statistically significant extent. Furthermore, a statistically significant negative association was observed between FMD and OSA severity.

**Table 1:** Clinical and metabolic characteristics of patients with OSA by severity and control snorers

	**OSA**			
	**snorers**	**mild/moderate**	**severe**	
	**AHI<5 n=47**	**AHI 5 – 29 n=61**	**AHI≥30 n=30**	**P**
**Age**	51.3±11.7	53.2±11.7	56.6±9.8	NS
**Males** (%)	66.0	70.5	83.3	NS
**AHI** (events/h)	1.2±1.4	14.6±8.0	42.8±14.2	<0.001
**Average SaO**_**²**_	95,0±1,4	93,6±2,2	92,6±2,5	<0,01
**Body Mass Index** (kg/m^2^)	29,3±3,9	30,5±4,5	32,8±5,1	<0,01
**Waist circumference** (cm)	102,5±11,1	105,8±12,1	113,6±12,1	<0,001
**Hip circumference** (cm)	106,5±8,5	109,9±9,1	116,9±11,7	<0,001
**Blood glucose** (mg/dl)	103.1±24.5	107.5±41.5	107.1±28.1	0.428
**Insulin** (μU/mL)	14.0±6.7	19.2±19.3	24.4±22.4	<0.05
**HOMA-IR**	3.6±1.9	5.8±6.8	8.4±11.1	<0.01
**Total cholesterol** (mg/dl)	210.4±41.4	201.4±38.7	207.9±39.9	NS
**LDL-cholesterol** (mg/dl)	137.7±35.2	128.0±33.7	128.8±33.3	NS
**HDL-cholesterol** (mg/dl)	46.8±11.5	46.0±11.5	45.8±9.9	NS
**Triglycerides** (mg/dl)	131.1±65.8	136.1±80.7	167.5±134.0	NS
**Urinary 8-iso-PGF2α** (pg/mg creatinine)	284.0±77,3	289.5±77,0	337.6±74.5	<0.001
**sNOX2-dp** (pg/ml)	26.2±8,2	26.3±7.5	28,8±8.0	NS
**Serum NOx** (uM/ml)	27.1±14.6	27.0±12,5	23,6±16.0	NS
**FMD%**	6.2±3.2	7.2±4.6	4.9±2.7	<0.05

Table 2 shows the prevalence of MS and its components in patients with different severity of OSAS. A statistically significant increase in the prevalence of central obesity was observed from snorers to subjects with severe OSAS (p=0.012), although a positive trend was also observed for the other components of MS. Fifty-six percent of patients with OSAS had MS, and 62.6%, 30.0% and 67.1% had hypertension, hyperlipidemia and hyperglycemia and/or type 2 diabetes, respectively.

**Table 2:** Prevalence of MS and its components in patients with different severity of OSAS

	**OSA**			
	**snorers**	**mild/moderate**	**severe**	
	**AHI<5 n=47**	**AHI 5 – 29 n=61**	**AHI≥30 n=30**	**P**
Hyperglycemia*	46	41	56,7	NS
Hypertension*	51,1	60,7	66,7	NS
Central obesity*	78,7	75,4	96,7	<0.05
Low HDL-cholesterol*	27,7	29,5	40	NS
Hypertriglyceridemia*	21,3	23	40	NS
All Metabolic syndrome*	46,8	49,2	70	NS
Metabolic syndrome* with diabetes	8,5	23	16,7	NS
**SCORE Metabolic Syndrome**	2.1±1.2	2.3±1.4	2.9±1.2	<0.05

*according to IFD criteria (Ref. [34]).

Table 3 shows bivariate correlations between AHI and some clinical and metabolic characteristics. AHI was significantly correlated with the indices of central obesity and with urinary 8-isoprostanes (r=0.298, p<0.001). In turn, urinary 8-iso-PGF2α were positively correlated with sNOX2-dp (r=0.250, p<0.01) and negatively correlated with NOx (r=0.360, p<0.001). A negative correlation was also observed between FMD and waist circumference (r=-0.199; p<0.05), serum insulin and triglyceride levels (r=-0.241; p=0.01 and r=-0.238; p<0.01 respectively) and the metabolic score (r=-0.335; p<0.001).

**Table 3:** Correlations between AHI and some clinical and metabolic characteristics

	**AHI**	
	**r**	**P**
**Age**	0.132	NS
**Average SaO**_**2**_	-0.386	<0,01
**Body Mass Index** (kg/m^2^)	0.271	<0,01
**Waist circumference** (cm)	0.294	<0.001
**Hip circumference** (cm)	0.295	<0.001
**Blood glucose** (mg/dl)	0.015	NS
**Insulin** (μU/mL)	0.249	<0.01
**HOMA-IR**	0.284	<0.01
**Total cholesterol** (mg/dl)	-0.034	NS
**LDL-cholesterol** (mg/dl)	-0.102	NS
**HDL-cholesterol** (mg/dl)	-0.055	NS
**Triglycerides** (mg/dl)	0.056	NS
**Urinary 8-iso-PGF2α** (pg/mg creatinine)	0.298	<0.001
**sNOX2-dp** (pg/ml)	0.013	NS
**Serum NOx** (uM/ml)	-0.135	NS
**FMD%**	-0.166	NS
**Metabolic score**	0.159	NS

In a stepwise multiple regression analysis including age, gender, waist circumference, AHI, Sa0_2,_ HOMA-IR, total cholesterol, systolic blood pressure, diabetes, urinary 8-iso-PGF2α and MS, as independent variable, MS (β= -0.40, t=-4.63, p<0.001 ) and urinary 8-iso-PGF2α (β= -0.17, t=-2.02, p<0.05) were the only independent predictors of FMD (R^2^=0.18).

Changes before and after CPAP treatment observed in the first consecutive 10 patients with severe OSAS who were compliant to CPAP treatment over a period of six months. are shown in Table 4. During treatment, mean HAI of the patients was significantly decreased from 43.4±12.6 at baseline to 7.0±5.0 (p<0.001), while mean Sa0_2_ increased from 91.8±3.7 to 97.0±1.0 (p=0.001). A statistically significant decrease of mean serum sNOX2-dp (p<0.005) and of urinary 8-iso-PGF2α ( p<0.01) was observed, while serum NOx showed only a minor statistically non significant increase. A statistically significant increase of FMD was also observed (from 3.6% to 7.0%). During the six months treatment, no significant change in body weight or cardiovascular risk factors was observed.

**Table 4:** Changes before and after nCPAP treatment in 10 patients with severe OSAS

	**Baseline**	**After C-PAP**	**P**
**Body Mass Index** (kg/m^2^)	35.3±5.4	36.0±6.0	NS
**AHI** (events/h)	43.4±12.6	7±5	0.000
**ODI** (events/h)	35.8±23.5	2.8±1.6	0.002
**Average SaO**_**2**_ (%)	91.8±3.7	97±1	0.001
**Urinary 8-iso-PGF2α** (pg/mg creatinine)	350.5±43.3	269.6±48.4	0.007
**sNOX2-dp** (pg/ml)	38.2±7.4	29±3.2	0.003
**Serum NOx** (uM/ml)	23.9±3.23	27.3±5	NS
**FMD** (%)	3.64±4.2	7.0±4.7	0.015

## Discussion

In our study, patients with severe OSA syndrome presented increased systemic oxidative stress. To assess oxidative stress in vivo, we measured urinary 8-isoprostanes, which are reliable markers of lipoperoxidation and, for the first time, sNOX2-dp, a marker of NOX2 activation by blood cells, which is a ROS generating enzyme implicated in arterial function via oxidative-stress mediated NO inactivation [3,37,38].

In fact, we found that patients with OSAS had significantly higher 8-iso-PGF2α urinary levels than healthy controls and that the severity of OSA was significantly correlated with the oxidative stress. In addition, severe OSAS had a tendency towards higher serum sNOX2-dp and lower serum nitrate and nitrite (NOx), which are stable NO derivatives, reflecting overall NO production. In keeping with the above results, we found a statistically significant decreased FMD in patients with severe OSAS. At multivariate analysis, MS and urinary 8-iso-PGF2α were independent predictors of FMD suggesting that in patients with OSA oxidative stress promotes arterial dysfunction likely via NO biosynthesis and/or inactivation. Moreover, as already reported, we further confirmed the strong association between MS and endothelial dysfunction [4].

However, earlier studies of oxidative stress in OSAS provided conflicting results. Yamauchi et al. [39] found increased urinary 8-hydroxy-20-deoxyguanosine (8-OhdG) excretion in the severe OSA patients, Carpagnano et al. [10] observed elevated 8-isoprostane levels in the exhaled breath condensate in OSA patients and Dyugovskaya et al. [40] detected an increase in the production of ROS in OSA. Several other studies reported abnormal lipid peroxidation in OSAS [7,8] or reduced total antioxidant status [9]. Conversely, Wali et al. [41] found no differences in susceptibility of LDL to oxidative stress and Svatikova et al. [42] reported that patients with moderate – severe OSA did not have evidence for greater oxidative stress and lipid peroxidation than healthy normal subjects. Negative results were also obtained in a recent study by Lee et al. [6] where no significant difference in either oxidative stress or antioxidant status markers was observed among normal patients and those with moderate and severe OSA; in this study, oxidative stress was related to central obesity rather than intermittent hypoxia and waist-to- hip ratio was a significant independent variable of oxidized-LDL, glutathione peroxidase, total antioxidant status and superoxide-dismutase.

In our study patients with OSA had reduced FMD, a widely used non invasive method to measure endothelial dysfunction. This is in agreement with the results reported by Oflaz et al. [19] and Grebe et al [43] who found a significant reduction of FMD in patients with OSA without other co-morbidities compared to healthy subjects; moreover, in the last study, the administration of i.v. vitamin C improved endothelial function in OSAS patients, leading to an increase of FMD to a level comparable to that observed in the control group. Similar findings were obtained by Büchner et al. [26] who found that endothelial function in OSAS patients improved either after infusion of vitamin C or after nCPAP treatment.

Conversely, Kato et al. [44] found no differences in FMD between OSA patients free of other diseases and healthy controls, although endothelium-dependent vasodilation tested by use of forearm blood flow responses to intra-arterial infusion of acetylcholine showed a blunted vasodilation in OSA patients.

In studies conducted by Chung et al. [45], oxygen desaturation index was the only significant determinant of FMD. Finally, in keeping with the above studies, Ip et al. [46] demonstrated that subjects with OSA had lower FMD compared with subjects without OSA and that major determinants of FMD in OSA patients were AHI and age.

The above data raise the question whether OSA itself results in oxidative stress and arterial dysfunction or it is simply a consequence of metabolic comorbidities frequently associated to OSAS. In fact, in a previous paper, we provided evidence that MS patients have lower FMD and NOx serum levels and increased urinary 8-iso-PGF2α and serum sNOX2-dp values, as compared to controls [47]. Thus, we acknowledge that MS and several potential confounders may influence oxidative stress in OSAS.

In our study, the first 10 patients with severe OSAS who adhered to nCPAP therapy were revaluated. Long-term compliance to nCPAP therapy was found to be effective in reducing the levels of systemic oxidative stress. In fact, nCPAP therapy normalized urinary 8-iso-PGF2α and serum sNOX2-dp values even though there was no significant change in body weight or cardiovascular risk factors during the six months treatment.

Our findings are in keeping with the results of several uncontrolled studies showing that short and long term nCPAP reduces oxidative stress in OSAS patients [7-10,25,27,28].

Conversely, in a study of 41 moderate-severe OSAS without other diseases, 4 h treatment with nCPAP did not affect plasma levels of isoprostanes and other markers of oxidative stress [41]. Moreover, one night nCPAP therapy did not significantly change the susceptibility of LDL to peroxidation and the levels of the antioxidant enzymes [42].

In our study, FMD showed a statistically significant increase after nCPAP over a six months period. This is in keeping with recently reported data [23,31,32,47]. Moreover, FMD significantly improved 1 week and 4 weeks after nCPAP in 10 male OSA patients concomitantly with an increase of plasma NOx concentrations, and a correlation between the two was observed [12]. Finally, FMD and endothelial nitric oxide production increased whereas expression of NFkB and nitrotyrosine decreased in patients who adhered to CPAP >4 hours daily [48].

In addition, in keeping with the above results, an improvement of carotid intima thickness, an early sign of atherosclerosis, was also observed following four-months nCPAP therapy [33].

Strength of our study are, first, that in contrast to many previous studies performed in otherwise healthy OSAS, we included patients with concomitant chronic diseases such as obesity, metabolic syndrome, hypertension, type 2 diabetes, i.e. a representative sample of the real-world OSA population. Indeed, prevalence of MS in our severe OSAS was 70% and 67% had hypertension and none of these clinical features did change after nCPAP therapy. By contrast, most published papers refer to otherwise healthy OSAS, without cardiovascular comorbidity, poorly representative of the habitual overall OSA population. Therefore, this is the first study to demonstrate the efficacy of nCPAP therapy on oxidative stress and arterial dysfunction in OSA patients with cardiometabolic risk factors, independent from weight loss and risk factor management.

Secondly, we assessed oxidative stress by measuring urinary 8-isoprostanes, which are elevated in several metabolic and cardiovascular diseases and possibly involved in atherosclerosis development and progression. Thirdly, we measured for the first time sNOX2-dp, a marker of NOX2 activation, a member of the NADPH oxidase family which plays an important role in ROS production and the key mechanisms underlying the development of endothelial dysfunction and cardiovascular pathophisiology. Indeed, so far, a role of NADPH oxidase in oxidative and proinflammatory responses after hypoxia/reoxigenation patterns simulating severe sleep apnea oxygenation has been demonstrated in a murine model of sleep apnea [49].

Unattended home PSG should be considered as a major limitation of this study, although an excellent correlation between the results of attended PSG and home monitoring has been demonstrated [50]. Indeed, PSG and home sleep tests use the same respiratory equipment, pulse oximetry equipment, and movement and position sensors and data generated from each test is analyzed in the same manner. Home monitoring has also the ability to record in a natural sleep environment and patients are tested in the comfort and privacy of their home. Further limitation of the study is the rather small number of patients who were included in the nCPAP study. Finally, the low correlation coefficients between AHI index and markers of oxidative stress suggest caution in the interpretation of the results.

## Conclusions

In conclusion, the results of our study indicate that patients with OSAS have an increased oxidative stress and arterial dysfunction that are partially reversed by nCPAP treatment.

We believe that our findings, which support a strong interplay between OSAS, MS, oxidative stress and arterial dysfunction, may be relevant for a better understanding of the pathogenesis of cardiovascular disorders in patients with OSAS.

## Competing interests

The authors declare that they have no competing interests.

## Authors’ contribution

MDB participated in the design and coordination of the study; MF and MB performed PSG studies and participated in the design of the study; LL and LP performed FMD studies; FA performed clinical studies; TA contributed to clinical data collection and elaboration: RC carried out the immunoassays; FV reviewed and edited data; FA wrote manuscript/study design and performed the statistical analysis; All authors read and approved the final manuscript.

## Financial disclosure

All the Authors have no relevant financial interest in this manuscript.

## Pre-publication history

The pre-publication history for this paper can be accessed here:

http://www.biomedcentral.com/1471-2466/12/36/prepub
